# Supplementary figures and images for: Insights into the representativeness of biodiversity assessment in large reservoir through eDNA metabarcoding
Source: PLoS One. 2025 Jan 24;20(1):e0314210. doi: 10.1371/journal.pone.0314210 (PMC11761093; doi:10.1371/journal.pone.0314210)

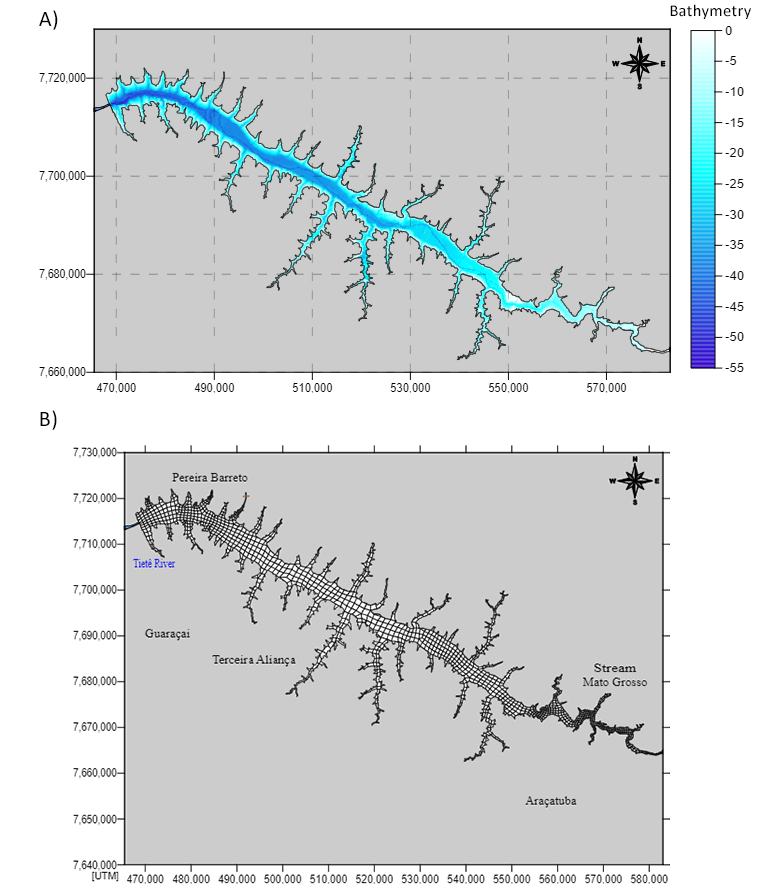

Supplement: S1 Fig — A) Bathymetry distribution across the reservoir. Darker shades show deeper sites. B) Biquadratic quadrangular finite elements for spatial discretization of the model. (PNG) [file pone.0314210.s001.png]

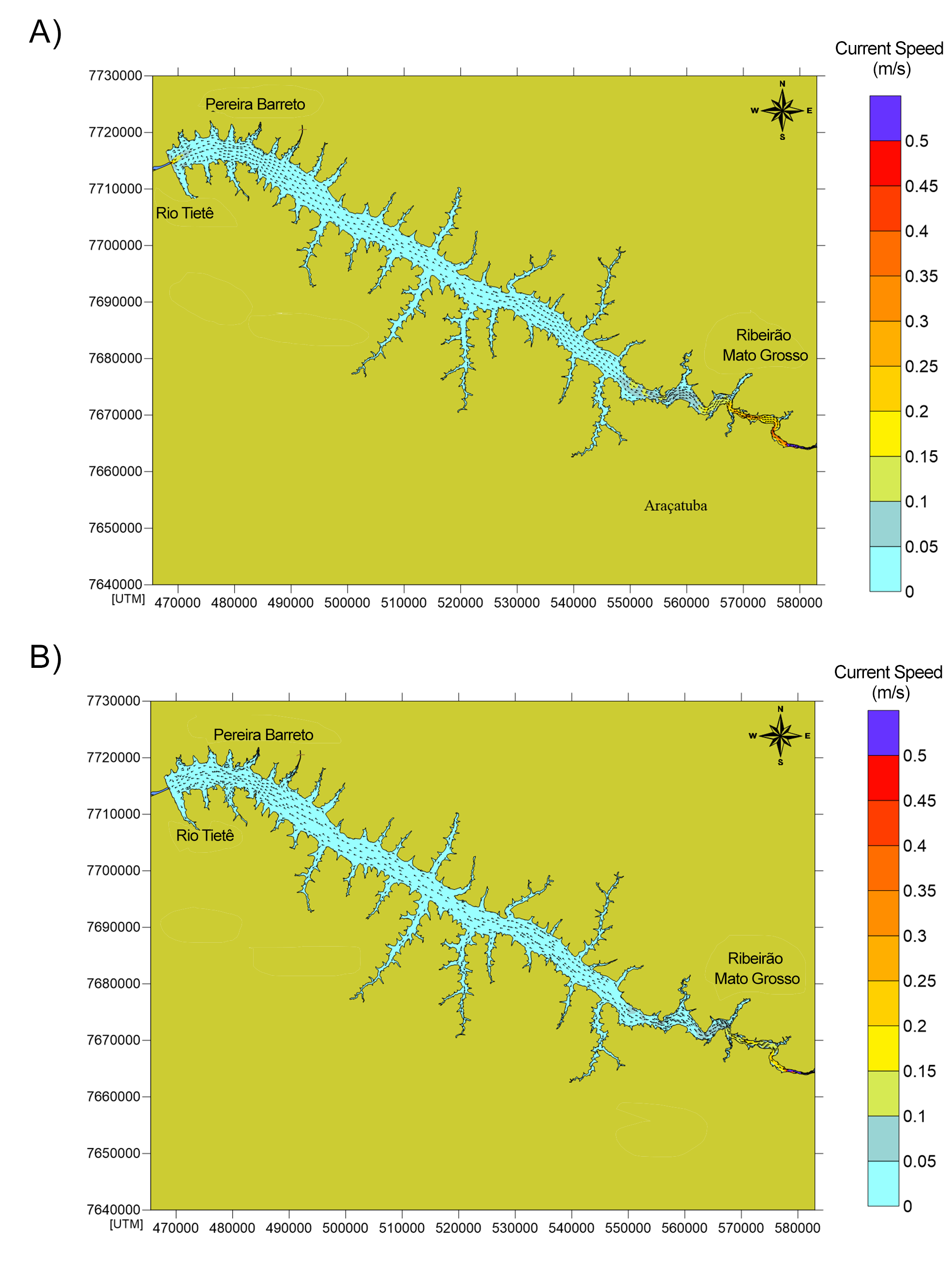

Supplement: S2 Fig — Maximum current speed as measured during the A) rainy and B) dry seasons. (PNG) [file pone.0314210.s002.png]

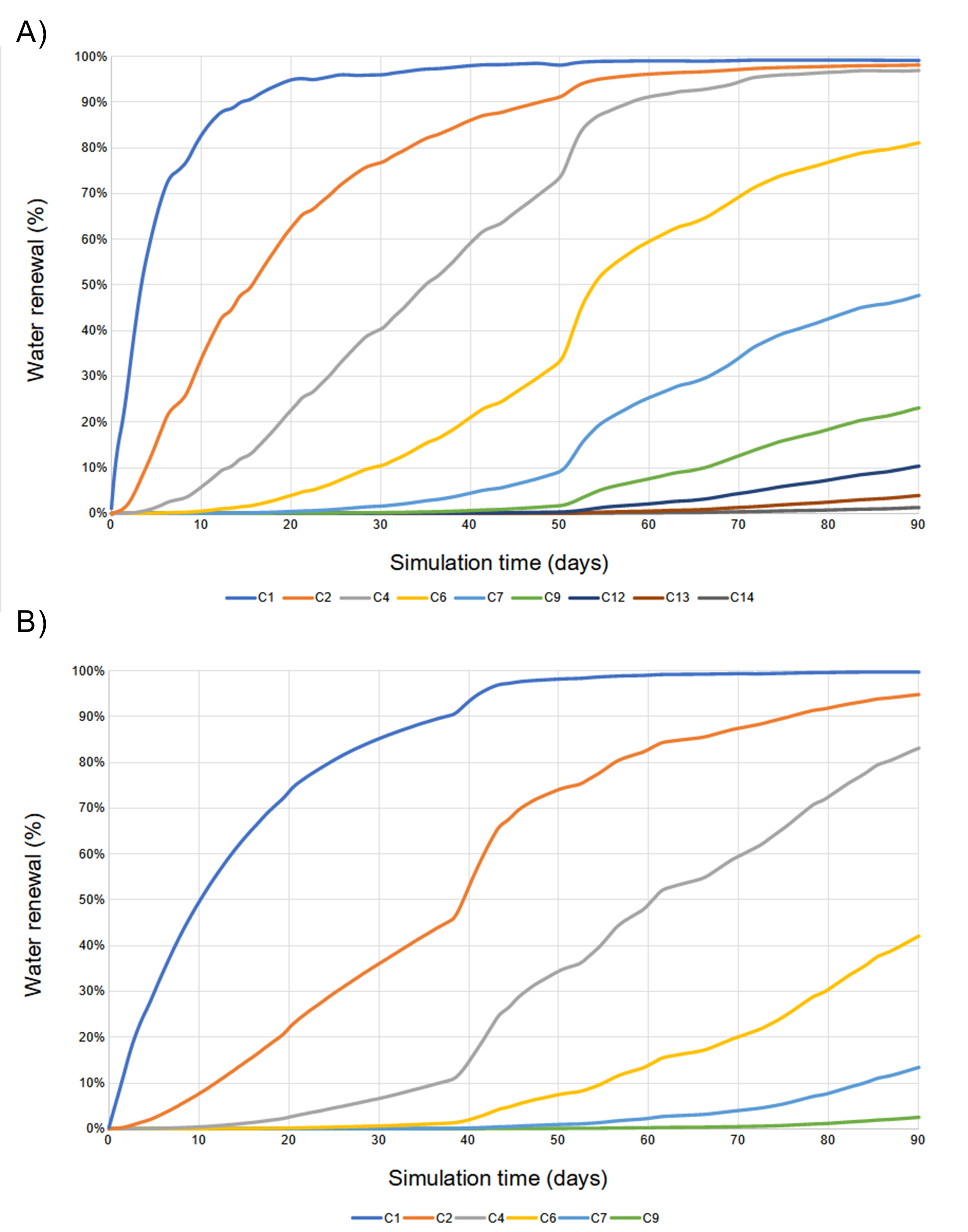

Supplement: S3 Fig — The water renewal is shown moving from Nova Avanhandava to Três Irmãos reservoir during A) rainy and B) dry scenarios. (PNG) [file pone.0314210.s003.png]

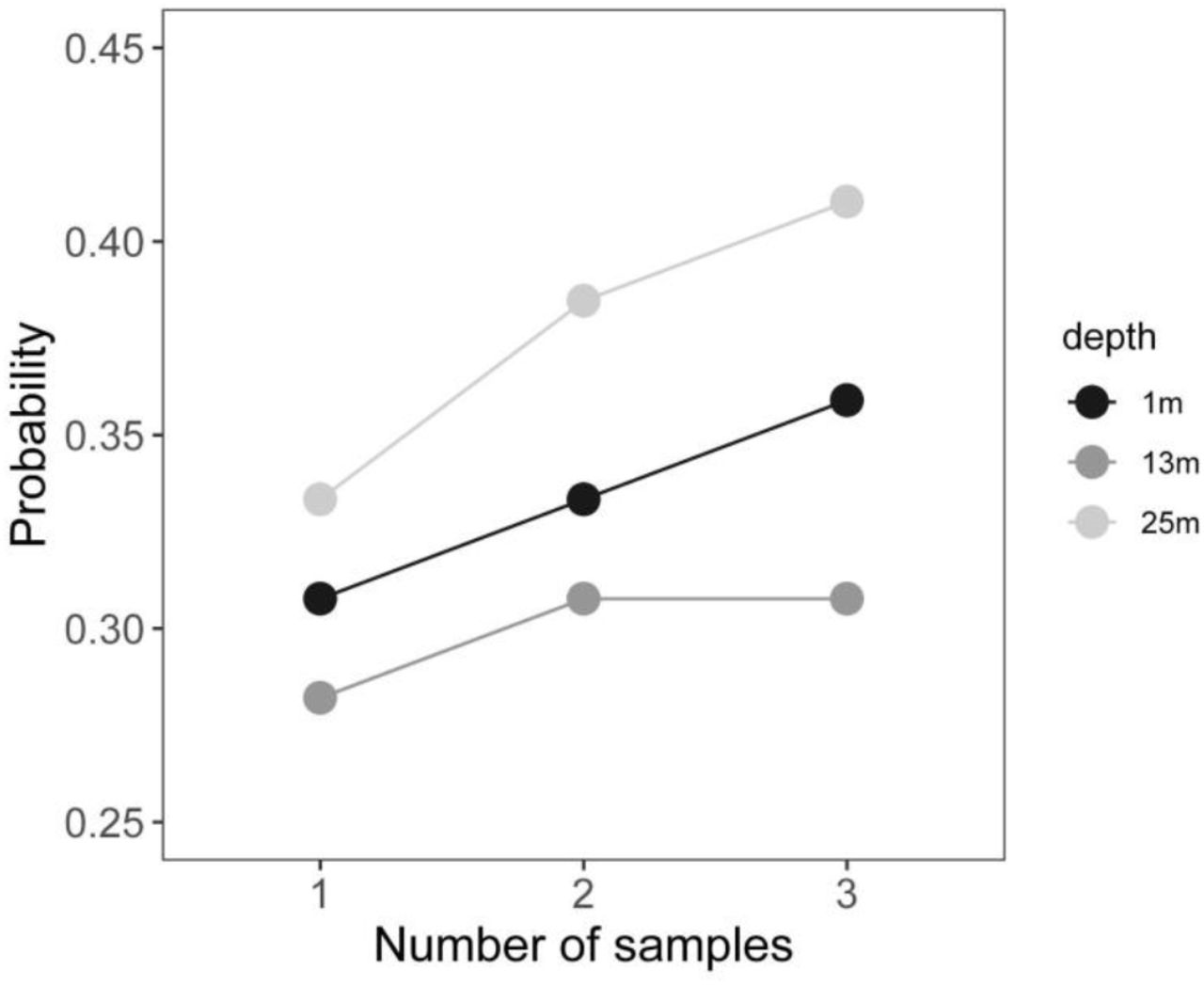

Supplement: S4 Fig — Generalized Linear Model (GLM) showing the probability (y-axis) of sampling fish taxa in different depths (1 meter, 13 meters and 25 meters from the surface) after three samples (x-axis). Each line represents one depth according to the colors in the legend. (PNG) [file pone.0314210.s004.png]

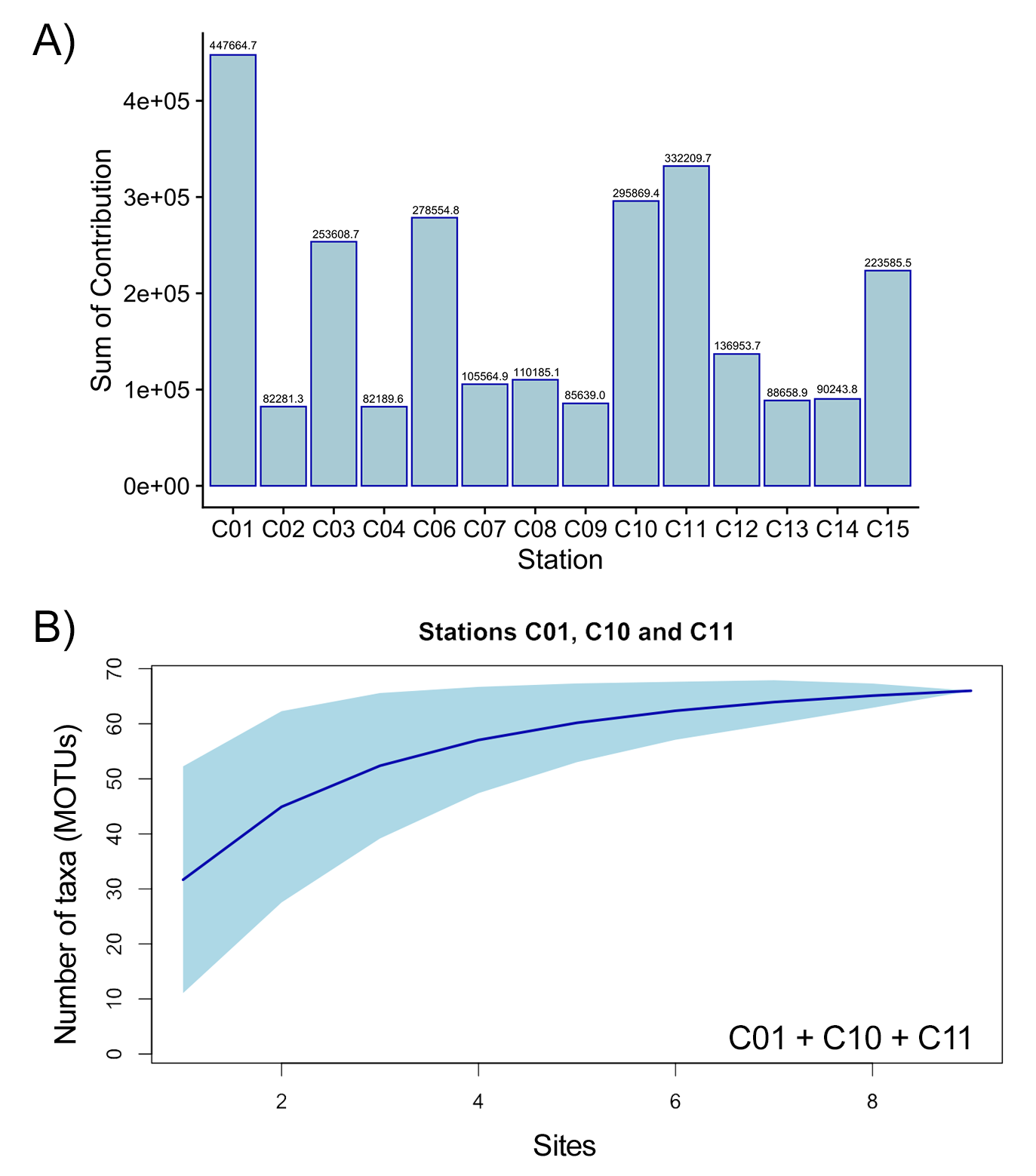

Supplement: S5 Fig — A) Sum of contribution of each sampled station for the DAPC clustering assignment. Given the differences in sampling size, the pilot sampling is not included. Station abbreviations as in S1 Table. Each bar corresponds to one station, and the value shows the sum of contribution for the six first Principal Components (see Methods). The stations with the highest values are C01, C11 and C10, respectively. B) Rarefaction curve constructed using only samples from C01, C11 and C10, where the curve reaches the plateau curve around eight samples. (PNG) [file pone.0314210.s005.png]
